# Ru Single Atoms Anchored on Oxygen‐Vacancy‐Rich ZrO_2‐x_/C for Synergistically Enhanced Hydrogen Oxidation

**DOI:** 10.1002/advs.202413569

**Published:** 2025-03-18

**Authors:** Xiaojuan Zhang, Chunchang Wang, Wenjun Cao, Qingqing Zhu, Chao Cheng, Jun Zheng, Haijuan Zhang, Youming Guo, Shouguo Huang, Yi Yu, Binghui Ge, Dongsheng Song, Yameng Fan, Zhenxiang Cheng

**Affiliations:** ^1^ Laboratory of Dielectric Functional Materials School of Materials Science & Engineering Anhui University Hefei 230601 China; ^2^ Institute of Physical Science and Information Technology Anhui University Hefei 230601 China; ^3^ Institute for Superconducting and Electronic Materials University of Wollongong Squires Way North Wollongong NSW 2500 Australia

**Keywords:** CO tolerant, density functional theory, hydrogen oxidation reaction, nanoparticles, Ru single‐atom catalysts

## Abstract

The hydrogen oxidation reaction (HOR) in alkaline media is pivotal for the advancement of anion exchange membrane fuel cells (AEMFCs), and the development of single‐atom catalysts offers a promising solution for creating cost‐effective, highly efficient HOR catalysts. Although the transition from nanoparticle to single‐atom catalysts enhances catalytic activity, the stability of these single‐atom sites remains a significant challenge. In this study, a highly active and stable alkaline HOR catalyst is successfully designed by incorporating Ru atoms into ZrO_2‐x_/C nanoparticles, forming the single atoms catalyst Ru‐SA‐ZrO_2‐x_/C. The catalyst exhibits an outstanding mass activity of 6789.4 mA mg_Ru_^−1^ at 50 mV, surpassing the Ru/C catalyst by 67 fold and the commercial Pt/C catalyst by 42.5 fold. Density functional theory (DFT) simulations reveal that the integration of Ru atoms into ZrO_2‐x_/C optimizes both the hydrogen bonding energy (HBE) and hydroxyl binding energy (OHBE), reducing the toxicity of Ru sites. This research opens a new pathway for the precise design of single‐atom and metal nanoparticle hybrids, offering a promising direction for developing highly active electrocatalysts for alkaline HOR applications.

## Graphical abstract

The alkaline HOR performance of ruthenium single atoms electrocatalysts are explored to investigate the catalytic action of ruthenium. Experimental characterization and theoretical simulations further demonstrate that low‐coordination Ru atoms modulate the electronic structure of the reaction site, optimizing hydrogen bonding energy and hydroxyl binding energy, which synergically accelerates HOR kinetics by lowering the reaction energy barrier.

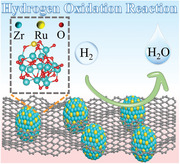

## 1 Introduction

With growing energy demands and escalating climate concerns, cleaner and more environmentally friendly renewable energy sources have garnered significant attention.^[^ ^1^ ^]^ Developing green and highly active electrocatalysts is critical for rapid energy conversion, as they lower the energy barrier and enhance the kinetics of electrochemical processes.^[^ ^2^ ^]^ Precious metal catalysts, such as Pt/C, Ir/C, and Ru/C, have significant advantages, but their commercial viability is hindered by scarcity and high costs.^[^ ^3^ ^]^ To address this problem, researchers have explored various strategies, including alloying, hybridization, and reducing nanoparticles to single atoms, to create efficient, stable, and cost‐effective electrocatalysts.^[^ ^4^ ^]^ Single‐atom catalysts (SACs) have attracted great interest in electrocatalysis due to their significant ability to enhance catalysis and selectivity.^[^ ^5^ ^]^ Designing metal nanoparticles into nanoclusters or single atoms can enhance these properties.^[^ ^6^ ^]^ However, the high surface free energy of isolated atoms makes them prone to aggregation. To stabilize these atoms, they are usually anchored on supports to form sturdy structures.^[^ ^7^ ^]^ Additionally, the interaction between the single atom and their carriers, as well as the unsaturated coordination environments of SACs, can further boost electrocatalytic performance by adjusting the electronic structure and reducing the reaction barrier.^[^ ^7^, ^8^ ^]^

The hydrogen Oxidation Reaction (HOR) is a crucial half‐reaction in renewable energy conversion technologies, such as fuel cells. However, the slow kinetics of HOR, especially in alkaline fuel cells, pose significant challenges.^[^ ^9^ ^]^ Recent research has focused on SAC‐based HOR catalysts that maintain efficiency and stability across a wide potential range, such as 3%Pt‐Ru/C, Co_1_Ru_1,n_/rGO, and Ru SA/WC_1−x_. These catalysts regulate the adsorption of H^*^ and OH^*^ reaction intermediates, accelerating HOR kinetics.^[^ ^4^, ^10^ ^]^ For instance, Peng and co‐workers developed a series of SACs dispersed on WC_x_ vectors (M‐WC_x_, M = Ru, Ir, Pd). In Ru‐WC_x_, the Ru single atoms form weak coordination bonds with W and C atoms, creating low‐valence active centers that optimize charge distribution and stabilize active sites. The synergistic interaction between these low‐valence Ru atoms and adjacent W sites helps to balance the adsorption of H_ad_ and OH_ad_, significantly lowering the thermodynamic energy barrier of the Volmer step.^[^ ^11^ ^]^ SACs are an ideal choice for theoretical studies in catalysis. However, carbon‐based single‐atom catalysts often exhibit unsatisfactory HOR performance in alkaline electrolytes, which seriously limits their application in alkaline membrane fuel cells. Therefore, developing efficient alkaline HOR catalysts based on SACs remains a major challenge.

Herein, we present an alkaline HOR electrocatalyst in which Ru atoms are introduced into oxygen‐vacancy‐rich ZrO_2‐x_/C nanoparticles, achieving highly efficient HOR activity. In an alkaline electrolyte, the Ru‐SA‐ZrO_2‐x_/C catalyst exhibits remarkable HOR activity, with a mass activity of 6789.4 mA mg_Ru_^−1^, which is 67 times higher than that of Ru/C (101.0 mA mg_Ru_^−1^) and 42.5 times higher than Pt/C (159.8 mA mg_Pt_^−1^). Density functional theory (DFT) calculations reveal that electron redistribution in the Ru‐SA‐ZrO_2‐x_/C structure, as well as enhanced OHBE and optimized HBE, are the reasons for the improved catalytic performance. This exceptional result underscores the potential of atomically dispersed metals in alkaline HOR electrocatalysis.

## 2 Results and Discussion

**Figure 1:**
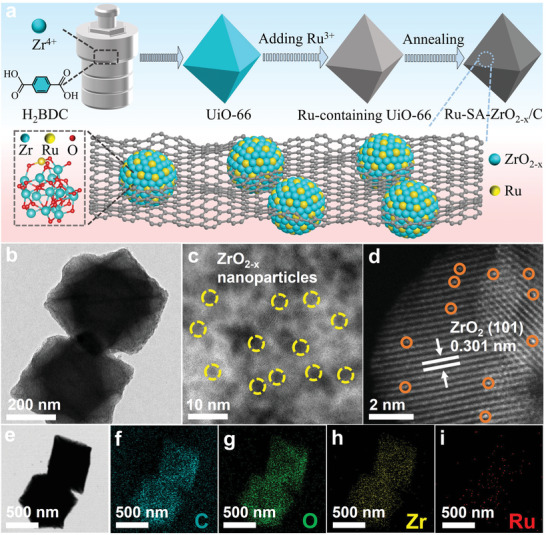
Structural characterization of Ru‐SA‐ZrO_2‐x_/C. a) Schematic of the preparation procedure for Ru‐SA‐ZrO_2‐x_/C. b) Low magnification TEM image of Ru‐SA‐ZrO_2‐x_/C. c) HRTEM image of Ru‐SA‐ZrO_2‐x_/C. d) HAADF‐STEM image of Ru‐SA‐ZrO_2‐x_/C. e–i) Elemental mappings of Ru, Zr, O, and C in Ru‐SA‐ZrO_2‐x_/C.

We successfully synthesized Ru single‐atom loaded ZrO_2‐x_/C nanoparticles (Ru‐SA‐ZrO_2‐x_/C) via a facile pyrolysis strategy under inert conditions. As illustrated in **Figure** **1**a, the precursor UiO‐66 was first synthesized with an octahedral morphology, as confirmed by X‐ray diffraction (XRD) and scanning electron microscope (SEM) (Figures S1 and S2, Supporting Information). Thermogravimetric analysis (TGA) (Figure S3, Supporting Information) indicated that the Ru‐SA‐ZrO_2‐x_/C catalyst was obtained by carbonizing the Ru‐containing UiO‐66 in an Ar atmosphere at 650 °C for 3 h. The catalyst retained the octahedral shape of UiO‐66 post‐calcination (Figure 1b; Figure S4, Supporting Information), confirming the robustness of the morphology throughout the synthesis. To further investigate the microstructure, high‐resolution transmission electron microscopy (HRTEM) and high‐angle annular dark‐field scanning transmission electron microscopy (HAADF‐STEM) were employed. As shown in Figure 1c and Figure S5 (Supporting Information), Ru‐SA‐ZrO_2‐x_/C appears as uniformly distributed nanoparticles (≈2.31 nm) embedded in the carbon matrix. The HAADF‐STEM image (Figure 1d) reveals distinct lattice fringes with a spacing of 0.30 nm, consistent with the interplanar distance of the (101) plane in ZrO_2_. Isolated bright spots (inside the orange circle) are identified as Ru atoms due to their high atomic number, confirming the successful loading of Ru atoms. The slight lattice expansion of ZrO_2_, as compared to pristine ZrO_2_, further corroborates the incorporation of Ru. STEM‐EDS measurements (Figure S6, Supporting Information) and ICP‐AES (Table S1, Supporting Information) give the Ru content of 0.42 wt.%. Elemental mappings (Figure 1e‐i) indicate a uniform distribution of C, O, Zr, and Ru in the catalyst, indicating atomic‐scale dispersion of Ru in ZrO_2‐x_ nanoparticles.

**Figure 2:**
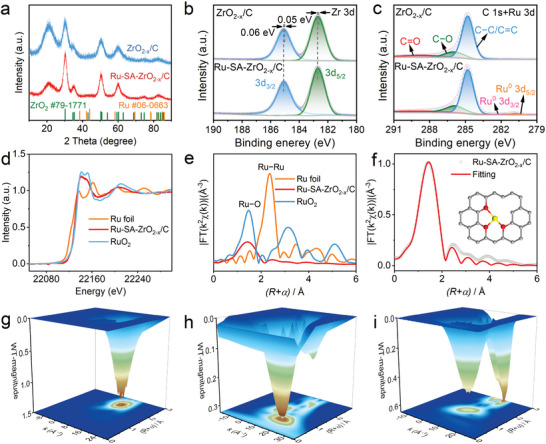
Fine structure analysis of Ru‐SA‐ZrO_2‐x_/C. a) XRD patterns of Ru‐SA‐ZrO_2‐x_/C and ZrO_2‐x_/C. XPS spectra of b) Zr 3d and c) C 1s and Ru 3d regions for Ru‐SA‐ZrO_2‐x_/C and ZrO_2‐x_/C, respectively. d) Ru K‐edge XANES spectra and e) FT‐EXAFS spectra of Ru foil, Ru‐SA‐ZrO_2‐x_/C, and RuO_2_. f) EXAFS fitting curves for Ru‐SA‐ZrO_2‐x_/C in R space. Wavelet transforms of the k^2^‐weighted EXAFS signal for g) Ru foil, h) Ru‐SA‐ZrO_2‐x_/C, and i) RuO_2_.

XRD patterns (**Figure** **2**a) of Ru‐SA‐ZrO_2‐x_/C and ZrO_2‐x_/C show diffraction peaks ≈30.3, 35.2, 50.3, and 60.2°, corresponding to the (101), (110), (112), and (211) planes of ZrO_2_, respectively (JCPDS 79–1771). The absence of Ru‐specific peaks suggests the atomic dispersion of Ru in the catalyst. Furthermore, samples prepared at different carbonization temperatures showed similar XRD patterns (Figure S7, Supporting Information), confirming that the temperature variation had no significant impact on the crystal structure. The specific surface area of Ru‐SA‐ZrO_2‐x_/C (282.2 m^2^ g^−1^), obtained from N_2_ adsorption–desorption isotherms, is significantly higher than that of ZrO_2‐x_/C (179.2 m^2^ g^−1^) (Figure S8, Supporting Information). This increase is attributed to defect‐rich structures induced by Ru atoms, which enhance mass transfer and expose more catalytic active sites. Electron paramagnetic resonance (EPR) (Figure S9, Supporting Information) analysis shows a strong asymmetric signal with a g‐factor of 2.003 for Ru‐SA‐ZrO_2‐x_/C, indicating the presence of oxygen vacancies, while the signal for ZrO_2‐x_/C was weaker. This suggests that there is a significant amount of local electron redistribution in Ru‐SA‐ZrO_2‐x_/C, enhancing its catalytic potential.

X‐ray photoelectron spectroscopy (XPS) was employed to analyze the surface chemical composition and electronic interaction between the single‐atomic Ru sites and the ZrO_2‐x_/C carrier. As shown in Figure S10 (Supporting Information), a weak Ru 3p signal appears in the spectrum after Ru loading, confirming the presence of Ru. In the Zr 3d region (Figure 2b), two strong peaks corresponding to Zr 3d_5/2_ and Zr 3d_3/2_ orbitals of Ru‐SA‐ZrO_2‐x_/C are observed at the binding energies of 182.7 and 185.1 eV, respectively.^[^ ^12^ ^]^ Notably, the positive shift in Zr 3d binding energy compared to ZrO_2‐x_/C indicates electron transfer from the ZrO_2‐x_/C carrier to the Ru single‐atoms site. The electron transfer suggests that ZrO_2‐x_/C acts as an electron donor, effectively reducing the valence state of Ru, promoting metal‐metal interactions, optimizing charge distribution around the single‐atom sites, and stabilizing the active sites. In the O 1s spectrum of Ru‐SA‐ZrO_2‐x_/C (Figure S11, Supporting Information), three distinct peaks are observed at the binding energies of 530.7, 531.72, and 533.35 eV, corresponding to surface lattice oxygen (O_L_), oxygen vacancy (O_v_), and surface hydroxyl group (O_OH_), respectively.^[^ ^13^ ^]^ Importantly, the oxygen vacancy content in Ru‐SA‐ZrO_2‐x_/C (49.5%) is significantly higher than that of ZrO_2‐x_/C (40.7%), indicating a higher concentration of defect sites that are critical for catalytic activity. In the C 1s spectrum, characteristic peaks at 284.8, 285.87, and 288.6 eV are attributed to C─C/C═C, C─O, and C═O bonds, in both ZrO_2‐x_/C and Ru‐SA‐ZrO_2‐x_/C, respectively (Figure 2c).^[^ ^14^ ^]^ In the Ru 3d spectrum of Ru‐SA‐ZrO_2‐x_/C, the binding energies of Ru 3d_5/2_ and Ru 3d_3/2_ are 280.8 and 281.6 eV, respectively, indicating that Ru is in a low‐valence state.^[^ ^15^ ^]^ Analysis of control samples (Figures S12 and S13, Supporting Information) further supports the presence of this low‐valence Ru. This finding confirms that Ru single atoms exhibit a low‐valence state, which is expected to enhance the electronic structure and catalytic performance of the material, making it an efficient catalyst.

X‐ray absorption near edge structure (XANES) and extended X‐ray absorption fine structure (EXAFS) spectroscopy were used to further analyze the local electronic structure and coordination environment of Ru single‐atoms sites. In the Ru K‐edge XANES spectrum, the average oxidation state of Ru in Ru‐SA‐ZrO_2‐x_/C falls between that of Ru foil and RuO_2_, suggesting that Ru in Ru‐SA‐ZrO_2‐x_/C is primarily in a metallic state (Figure 2d).^[^ ^15^, ^16^ ^]^ This means that the charge transfer between Ru single‐atoms and ZrO_2‐x_/C enhances the local electron configuration. The Fourier transform of the EXAFS (FT‐EXAFS) spectrum shows a distinct peak at ≈1.42 Å for Ru‐SA‐ZrO_2‐x_/C, indicating isolated Ru atoms coordinating with oxygen (Figure 2e). Notably, the characteristic RuO_2_ peaks at 3.13 Å were absent in the EXAFS spectrum, confirming the absence of RuO_2_ in Ru‐SA‐ZrO_2‐x_/C.^[^ ^17^ ^]^ Furthermore, no Ru─Ru bonding peaks were detected in the EXAFS spectrum, affirming the existence of Ru in an atomically dispersed form. Quantitative EXAFS curve fitting analysis of Ru‐SA‐ZrO_2‐x_/C was conducted to extract structural parameters. As shown in Figure 2f and Table S2 (Supporting Information), the coordination number of Ru─O is 3.1, indicating that each Ru atom is coordinated with ≈3 oxygen atoms (inset of Figure 2f). The Ru─O bond length was determined to be 2.05 Å, which is longer than the Ru─Ru bond length of 2.68 Å (Figures S14 and S15, Supporting Information), suggesting that the Ru─O coordination is thermodynamically more stable than Ru─Ru bonding. To further confirm the bonding environment of Ru single atoms, high‐resolution wavelet transforms (WT‐EXAFS) analysis was performed in both k and R space (Figure 2g–i). The WT diagram of the Ru foil shows a strong signal at k ≈ 10.2 Å^−1^, which is attributed to the Ru─Ru bond.^[^ ^18^ ^]^ In contrast, the WT diagram for RuO_2_ reveals two maximum signals corresponding to Ru─O and Ru─Ru bonds. The maximum WT intensity for Ru‐SA‐ZrO_2‐x_/C is at k ≈ 5.7 Å^−1^, further confirming the absence of Ru−Ru bonds and the coordination between Ru and O atoms.

**Figure 3:**
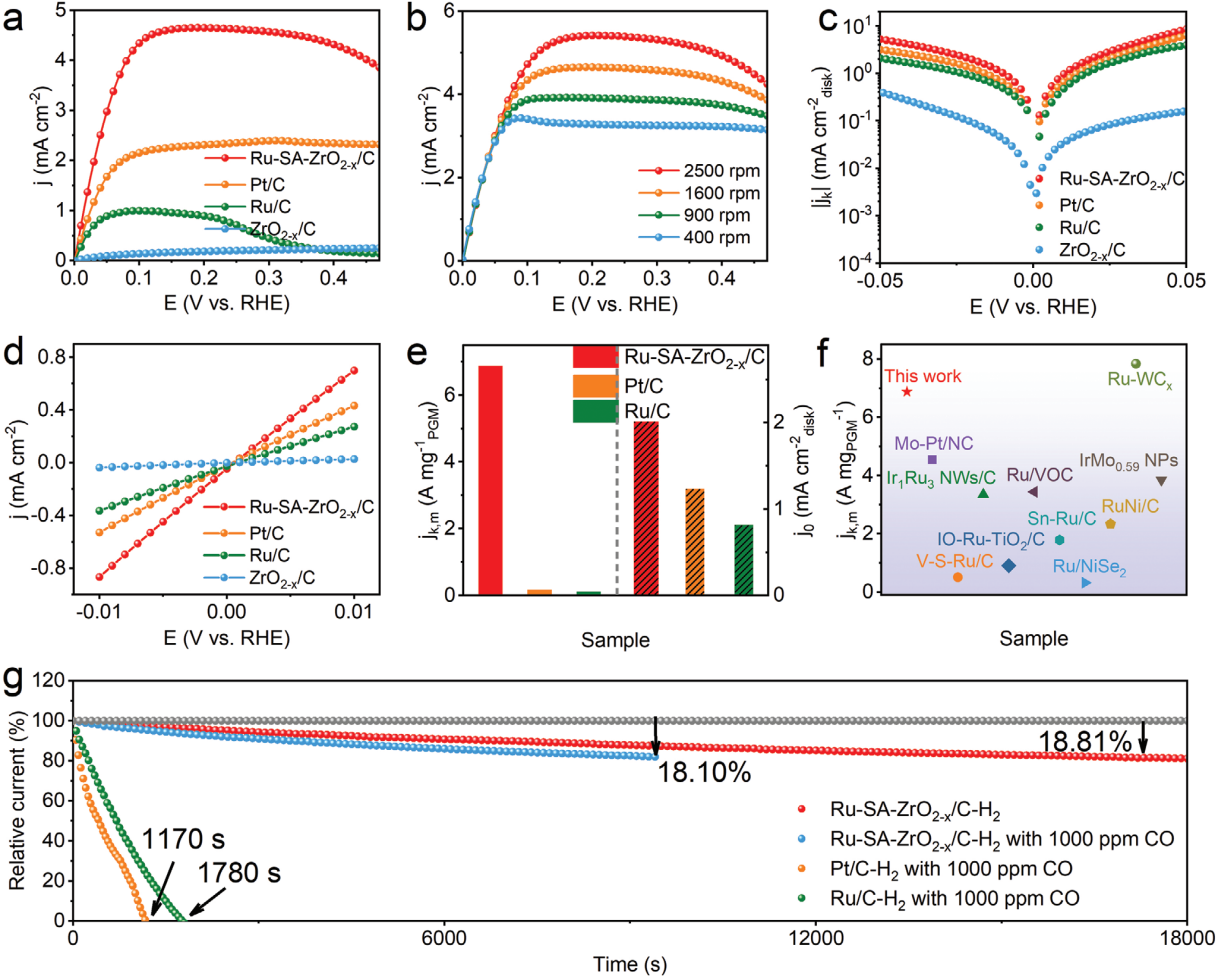
Electrochemical performance for alkaline HOR. a) HOR polarization curves of Ru‐SA‐ZrO_2‐x_/C, Pt/C, Ru/C, and ZrO_2‐x_/C in H_2_‐saturated 0.1 m KOH with a scan rate of 5 mV s^−1^ at 1600 rpm. b) HOR polarization curves of Ru‐SA‐ZrO_2‐x_/C at different rotation speeds. c) Tafel plots with solid lines representing Butler‐Volmer fitting. d) Linear fitting curves in micro‐polarization region (−10–10 mV). e) Comparison of mass activity at 50 mV and exchange current density (j_0_) for Ru‐SA‐ZrO_2‐x_/C, Pt/C, and Ru/C. f) Comparison of the j_k,m_ of Ru‐SA‐ZrO_2‐x_/C with noble metal‐based catalysts reported in the literature. g) Chronoamperometry responses of Ru‐SA‐ZrO_2‐x_/C, Pt/C, and Ru/C in 0.1 m KOH solutions.

Inspired by the unique electronic structure of the Ru‐SA‐ZrO_2‐x_/C catalyst, its electrochemical performance for HOR was evaluated using a typical three‐electrode system. Electrocatalytic measurements were performed with linear sweep voltammetry at a scanning rate of 5 mV s^−1^ in an H_2_‐saturated 0.1 m KOH electrolyte. Among the tested materials, Ru‐SA‐ZrO_2‐x_/C exhibits the highest HOR activity across the entire potential range (**Figure** **3**a), whereas ZrO_2‐x_/C shows almost no HOR activity. To further investigate the HOR kinetics, polarization curves for Ru‐SA‐ZrO_2‐x_/C were measured at various rotation speeds (400–2500 rpm). As the rotation speed increases, the current density also increases, indicating enhanced mass transport. To provide evidence for a dual‐electron transfer mechanism (Figure 3b), using the Koutecky–Levich equation, the calculated slope of Ru‐SA‐ZrO_2‐x_/C at an overpotential of 150 mV is 4.75 cm^2^ mA^−1^ s^−1/2^, which closely matches the theoretical value of 4.87 cm^2^ mA^−1^ s^−1/2^ for a two‐electron HOR process (Figure S16, Supporting Information).^[^ ^19^ ^]^ Furthermore, no HOR current is detected in an Ar‐saturated atmosphere (Figure S17, Supporting Information), confirming that the anode current in Ru‐SA‐ZrO_2‐x_/C originates from HOR. For a deeper understanding of the catalytic activity, we analyzed the dynamic current density (j_k_) using the Koutecky–Levich equation. As shown in Figure 3c, the j_k_ of Ru‐SA‐ZrO_2‐x_/C outperforms that of the commercial Pt/C across a range of potentials, reaching a maximum of 8.49 mA cm^−2^ at 50 mV overpotential. This demonstrates that the incorporation of Ru single‐atoms significantly improves the electronic structure and catalytic kinetics of HOR. The exchange current density (j_0_) was determined by linear fitting of the micropolarization region (Figure 3d). Ru‐SA‐ZrO_2‐x_/C exhibits a much higher j_0_ (2.01 mA cm^−2^) as compared to Pt/C (1.2 mA cm^−2^) and Ru (0.8 mA cm^−2^), underscoring its superior catalytic performance. For a fair comparison, mass‐normalized activity (j_k,m_) was also evaluated. At 50 mV, the j_k,m_ of Ru‐SA‐ZrO_2‐x_/C is 6789.4 mA mg_Ru_^−1^, which is 67 times that of Ru/C (101.0 mA mg_Ru_^−1^) and 42.5 times that of Pt/C (159.8 mA mg_Pt_^−1^), indicating that the utilization efficiency of Ru single atoms in Ru‐SA‐ZrO_2‐x_/C is much higher (Figure 3e). In addition, the HOR performance of a series of catalysts synthesized at different pyrolysis temperatures was compared (Figure S18, Supporting Information). As shown in Figure 3f and Table S3 (Supporting Information), the mass activity of the Ru‐SA‐ZrO_2‐x_/C ranks the highest level among the reported noble‐metal based HOR catalysts, further demonstrating its outstanding catalytic efficiency.

The stability of the HOR catalyst is a crucial factor in evaluating the real‐world performance of AEMFCs.^[^ ^20^ ^]^ To access this, we performed chronoamperometry to examine the long‐term stability of the catalysts. As shown in Figure 3g, the relative current density of the Ru‐SA‐ZrO_2‐x_/C decreases by only 18.81% after 18 000 s of continuous operation in a hydrogen‐saturated electrolyte, demonstrating excellent stability. CO tolerance is another critical factor for the practical application of catalysts.^[^ ^21^ ^]^ We tested the HOR activity of various catalysts in the presence of 1000 ppm CO. The Ru‐SA‐ZrO_2‐x_/C exhibits minimal decay, maintaining 81.9% of its activity after 9500 s of continuous cycling. In sharp contrast, commercial Pt/C and Ru/C usually show significant activity loss, with Pt/C nearly losing all activity at 1170 s and Ru/C losing all activity at 1780 s. This indicates that the active sites in these catalysts are severely poisoned by CO, whereas the Ru‐SA‐ZrO_2‐x_/C exhibits excellent resistance to CO poisoning. The enhanced CO tolerance of Ru‐SA‐ZrO_2‐x_/C may be due to the finely tuned electronic structure, which reduces ΔE_CO_. This modification ensures a uniform distribution of OH^*^ spices around Ru atoms on the catalyst surface, allowing rapid oxidation of CO and thereby preventing poisoning of the active sites. After the durability test, the morphology and structural integrity of Ru‐SA‐ZrO_2‐x_/C hardly changes (Figures S19–S21, Supporting Information), indicating excellent structural stability and corrosion resistance. As expected, the precise regulation of the electronic structure in the Ru single‐atom sites of Ru‐SA‐ZrO_2‐x_/C endows the catalyst with outstanding HOR electrochemical activity and durability.

**Figure 4:**
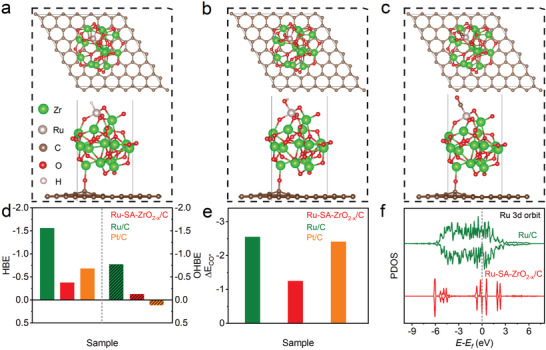
Theoretical analysis. Atomic configuration of Ru‐SA‐ZrO_2‐x_/C with adsorbed a) H^*^, b) OH^*^, and c) CO^*^ on the Ru site, shown from both the side view and the top view. d) Calculated optimal binding energies (HBEs and OHBEs) and e) CO adsorption energy on Ru, Ru‐SA‐ZrO_2‐x_/C, and Pt models. f) The PDOS of Ru atoms in Ru‐SA‐ZrO_2‐x_/C and Ru cluster.

Density functional theory (DFT) calculations further elucidate how the incorporation of Ru single‐atom into ZrO_2‐x_/C enhances alkaline HOR activity and provides excellent CO tolerance. Based on the experimental data, an interfacial model of single‐atom Ru doped into ZrO_2‐x_/C clusters (Ru‐SA‐ZrO_2‐x_/C) was constructed on graphene. For comparison, models for Ru and Pt clusters were also created. We calculated the possible adsorption sites for H^*^, OH^*^, and CO^*^ species, with the optimal adsorption sites shown in Figures S22–S24 (Supporting Information). From a thermodynamic perspective, we first analyzed the sites at the Ru‐SA‐ZrO_2‐x_/C interface, focusing on both the Ru and ZrO_2‐x_/C sides. **Figure** **4**a–c displays the optimal adsorption sites for H*, OH*, and CO* at the Ru active sites near the ZrO_2‐x_/C clusters. Notably, the hydrogen and hydroxide binding energies (HBE and OHBE) at the Ru‐SA‐ZrO_2‐x_/C interface are weaker than those at the Ru clusters. The HBE of Ru‐SA‐ZrO_2‐x_/C is comparable to that of Pt (Figure 4d), while the OHBE is moderate, which helps to protect the metal from oxidation and maintains the activity and stability of the catalyst at high potentials. Ruthenium is considered a potential HOR catalyst due to its strong oxygen affinity.^[^ ^22^ ^]^ However, pure Ru exhibits excessively strong hydroxyl adsorption and is prone to oxidation, making it a suboptimal choice for alkaline HOR catalysts.^[^ ^21^, ^23^ ^]^ Therefore, an ideal alkaline HOR catalyst should possess weaker HBE and OHBE than pure Ru. Pt, while effective at adsorbing H^*^, has too low an OHBE to provide sufficient OH^*^ adsorption sites, leading to poor alkaline HOR activity. In contrast, Ru‐SA‐ZrO_2‐x_/C shows stronger OHBE than Pt clusters, promoting the removal of adsorbed CO (CO_ad_) and improving CO tolerance. Figure 4e illustrates the CO adsorption energy (E_CO*_) for different catalysts. The E_CO*_ for Ru and Pt clusters are significantly higher than that of Ru‐SA‐ZrO_2‐x_/C, indicating that CO binds more weakly to Ru in Ru‐SA‐ZrO_2‐x_/C. This weak CO adsorption helps to prevent poisoning of the Ru single‐atom site, confirming the excellent anti‐CO poisoning capability of Ru‐SA‐ZrO_2‐x_/C. The state density (DOS) near the Fermi level is directly related to the adsorption energy of intermediates. We calculated the project DOS (PDOS) of Ru atoms in the Ru cluster and Ru‐SA‐ZrO_2‐x_/C (Figure 4f). The D‐band center of Ru in the Ru cluster is located at −1.5 eV, while in the Ru‐SA‐ZrO_2‐x_/C model, the D‐band center shifts to −2.47 eV, indicating a higher energy state. This shift weakens the adsorption of H^*^ and OH^*^ at the Ru interface. Overall, the incorporation of Ru single atoms into ZrO_2‐x_/C optimizes HBE and OHBE at the interface, reduces OH^*^ adsorption at the metal site, and generates a synergistic effect that enhances both HOR activity and catalyst stability.

## 3 Conclusion

In summary, we have developed a novel approach that significantly improves the alkaline HOR activity and CO tolerance by incorporating Ru single atoms into ZrO_2‐x_/C nanoparticles. The Ru‐SA‐ZrO_2‐x_/C electrocatalyst was found to exhibit enhanced conductivity and full of abundant oxygen vacancies. The interaction between the Ru single atoms and the ZrO_2‐x_/C nanoparticles facilitates electron redistribution and creates highly active catalytic sites, leading to improved catalytic performance. Theoretical simulations further demonstrate that low‐coordination Ru atoms modulate the electronic structure of the reaction site, optimizing HBE and OHBE, which synergically accelerates HOR kinetics by lowering the reaction energy barrier. The Ru‐SA‐ZrO_2‐x_/C shows exceptional long‐term stability and excellent CO tolerance, achieving an impressive mass activity of 6789.4 mA mg_Ru_^−1^ at 50 mV, outperforming both Ru/C catalyst and commercial Pt/C catalysts. This study presents a promising strategy for designing catalysts with maximal atomic efficiency and offers valuable insights for developing cost‐effective, high‐performance AEMFC technologies.

## 4 Experimental Section

Detailed experimental procedures can be found in the Supporting Information.

## Conflict of Interest

The authors declare no conflict of interest.

## Supporting information



Supporting Information

## Data Availability

The data that support the findings of this study are available from the corresponding author upon reasonable request.
