# Supplementary material for: Ru Single Atoms Anchored on Oxygen‐Vacancy‐Rich ZrO2‐x/C for Synergistically Enhanced Hydrogen Oxidation
Source: Adv Sci (Weinh). 2025 Mar 18;12(18):2413569. doi: 10.1002/advs.202413569 (PMC12079528; doi:10.1002/advs.202413569)
Supplement: Supplementary file 1 — Supporting Information [file ADVS-12-2413569-s001.docx]

**Supporting Information**

# Ru Single Atoms Anchored on Oxygen-Vacancy-Rich ZrO_2-x_/C for Synergistically Enhanced Hydrogen Oxidation

*Xiaojuan Zhang^1^, Chunchang Wang^1^*, Wenjun Cao^1^, Qingqing Zhu^1^, Chao Cheng^1,2^, Jun Zheng^1,2^, Haijuan Zhang^1^, Youming Guo^1^, Shouguo Huang^1^, Yi Yu^1^, Binghui Ge^2^, Dongsheng Song^2^*, Yameng Fan^3^, Zhenxiang Cheng^3^**

^1^Laboratory of Dielectric Functional Materials, School of Materials Science & Engineering, Anhui University, Hefei 230601, China

^2^Institute of Physical Science and Information Technology, Anhui University, Hefei 230601, China

^3^Institute for Superconducting and Electronic Materials, University of Wollongong, Squires Way, North Wollongong, NSW 2500, Australia

*Corresponding author, E-mail: ccwang@ahu.edu.cn (WANG), [dsong@ahu.edu.cn](mailto:dsong@ahu.edu.cn) (SONG), [cheng@uow.edu.an](mailto:cheng@uow.edu.an) (CHENG)

**1. Experimental section**

**1.1 Material synthesis**

All chemicals were of analytical grade and used without further purification.

***1.1.1 Synthesis of the UiO-66***

The preparation of UiO-66 octahedrons was based on a reported method with some modifications. Typically, 80 mg of H_2_BDC, 120 mg of ZrCl_4_ and 8 mL of acetic acid were completely dissolved in 40 mL of N,N-dimethylformamide (DMF) using ultrasonic treatment. The mixture was transferred to a Teflon reactor inside a stainless‑steel autoclave and heated at 120 ºC for 12 h in a programmable oven under static condition. After cooling down to room temperature, the precipitate was washed sequentially with DMF and deionized water by centrifugation, and finally dried in air at 60 °C overnight.

***1.1.2 Synthesis of the Ru-SA-ZrO_2-x_/C***

50 mg of the resulting UiO-66 was dispersed in 15 mL of ethanol and sonicated to obtain a homogeneous mixture. Then, 5 mL of deionized water containing 10 mg of RuCl_3_ was added to the suspension and stirred for 10 h in the dark. The product was collected and dried in an oven at 60 °C overnight. Ru-SA-ZrO_2-x_/C samples were obtained by thermally annealing the synthesized materials under an Ar atmosphere at temperatures ranging from 600 to 700 °C for 3 hours with a ramping rate of 2 °C min^−1^.

***1.1.3 Synthesis of the ZrO_2-x_/C***

The UiO-66 was carbonized at 650 °C for 3 h with a heating rate of 2 °C min^−1^ in an Ar atmosphere.

**1.2 Material characterization**

The crystal phases of all samples were analyzed suing a power X-ray diffractometer (SmartLab9KW) equipped using Cu-Kα radiation. The morphologies of the as-prepared samples are investigated using scanning electron microscopy (SEM, JEOL JSM-6700 M) at 20 kV and transmission electron microscopy (TEM, Hitachi H-800) at 200 kV. High resolution transmission electron microscopy (HRTEM, JEOL-2011) was used to examine the structure of the samples. High-angle annular dark field-scanning transmission electron microscopy (HAADF-STEM) images were acquired by using a double spherical aberration correction transmission electron microscope (Titan Themis Z, 300 kV). Thermal stability and sulfur loading mass were characterized using a TGA-5500 thermoanalyser (TGA) under flowing nitrogen with a heating rate of 10 ℃ min^−1^. X-ray photoelectron spectroscopy (XPS, ESCALAB 250 Xi) was used to study chemical valence and compositions. Specific surface area and pore structural characteristics were measured using a sorption analyzer (Micromeritics ASAP 2020) at 77 K, with pore volume and size calculated using the Barrett-Joyner Halenda (BJH) method. Raman spectrum were obtained using a Confocal Laser Micro Raman Spectrometer (Via-Reflex/inVia-Reflex). Ru K-edge X-ray absorption near edge structure (XANES) data was collected at the Synchrotron Light Source Laboratory in Australia.

**1.3 Electrochemical measurements**

2.0 mg of samples were mixed with 0.5 mL of ethanol containing 3.0 wt.% Nafion and ultrasonicated for more than 30 minutes to form a homogeneous ink. The glassy carbon (GC, 5 mm in diameter) electrode was polished using 1.0, 0.5 and 0.05 *μ*m *γ*-Al_2_O_3_ powder slurry washed with ultrapure water and ethanol via sonication and air-dried. 15 *μ*L of the ink was drop-casted on the GC electrode and air-dried before electrochemical measurements. The optimal catalyst loading on the GC rotating disk electrode (RDE) was determined to be 0.297 mg cm^−2^.

Electrochemical tests were conducted at CHI 760E electrochemistry workstation. The electrocatalyst-coated GC electrode was the working electrode, with a platinum mesh as the counter electrode and an Ag/AgCl electrode (full filled with 3.0 M KCl) as the reference. All measured potentials were referred to the reversible hydrogen electrode (RHE) potential. For each test, fresh 0.1 M KOH was used as the electrolyte.

Before hydrogen oxidation reaction (HOR) evaluation, the GC disk surface was stabilized at open circuit potential for over 300 s in H_2_-saturated 0.1 M KOH solution. Polarization curves were recorded using a rotating disk electrode (RDE) system (Pine Research Instruments). HOR performance was tested in H_2_-saturatued 0.1 M KOH with the RDE rotating at speeds from 400 to 2500 rpm at a scan rate of 5 mV s^−1^. Kinetic current density (j_k_) was extracted from the Koutecky-Levich equation (Eq. S1),
 $\frac{1}{j}=\frac{1}{j_{k}}+\frac{1}{j_{d}}=\frac{1}{j_{k}}+\frac{1}{Bc_{0}\omega^{1/2}}$ **Eq. S1**

where j, j_d_, B, c_0,_ and ω *represent* the measured current density, diffusion-limited current density, the Levich constant, the solubility of H_2_ (7.33 × 10^−4^ mol L^−1^), and the rotation speed, respectively. B is calculated from Eq. S2,

$B=0.2nFD^{2/3}\nu^{-1/6}$ **Eq. S2**

where n is the number of electrons transferred, F is the Faraday constant (96485 C mol^-1^), D is the diffusivity of H_2_ (3.7 × 10^−5^ cm^2^ s^−1^), and ν is the kinematic viscosity (1.01×10^−2^ cm^2^ s^−1^), respectively.

Exchange current density (j_0_), a measure of catalytic intrinstic activity, was calculated using the Butler-Volmer equation (Eq. S3),

$j_{k}=j_{0}\left[ e^{\frac{\alpha F\eta}{\mathrm{RT}}}-e^{\frac{-(1-\alpha)F\eta}{\mathrm{RT}}} \right]$ **Eq. S3**

where α represent the transfer coefficient, R is the universal gas constant (8.314 J mol^−1^ K^−1^), T is the operating temperature (298.15 K in this work), and η is the overpotential.

**1.4 Calculation details**

All calculations were based on density functional theory using the Vienna Ab-initio Simulation Package (VASP).^[1]^ The generalized gradient approximation (GGA) with Perdew-Becke-Ernzerh (PBE) function was employed to describe the electron interaction energies for exchange correlation.^[2]^ The projector augmented wave method was applied for electron-ion interactions, with a plane-wave energy cutoff of 450 eV. A Monkhorst-Pack k-points grid of 3$\times3\times$1 was used for all the calculations, and the vacuum region was set to 15$Å$ in z direction to prevent the interaction between two adjacent surfaces. The energy convergence criterion was set to 10^−5^ eV.

Adsorption energies (*E_ads_*) was calculated using the following equation,

*E_ads_* = *E_tot_* $-$ *E_surf_* $-$ *E_gas_* **Eq. S4**

where *E_tot_* is the total energies of surface with adsorption, *E_surf_* is the surface energy without adsorption, and *E_gas_* is the gas-phase energy for H_2_ and H_2_O.





**Figure S1.** XRD patterns of the synthesized UiO-66 and the simulated UiO-66.


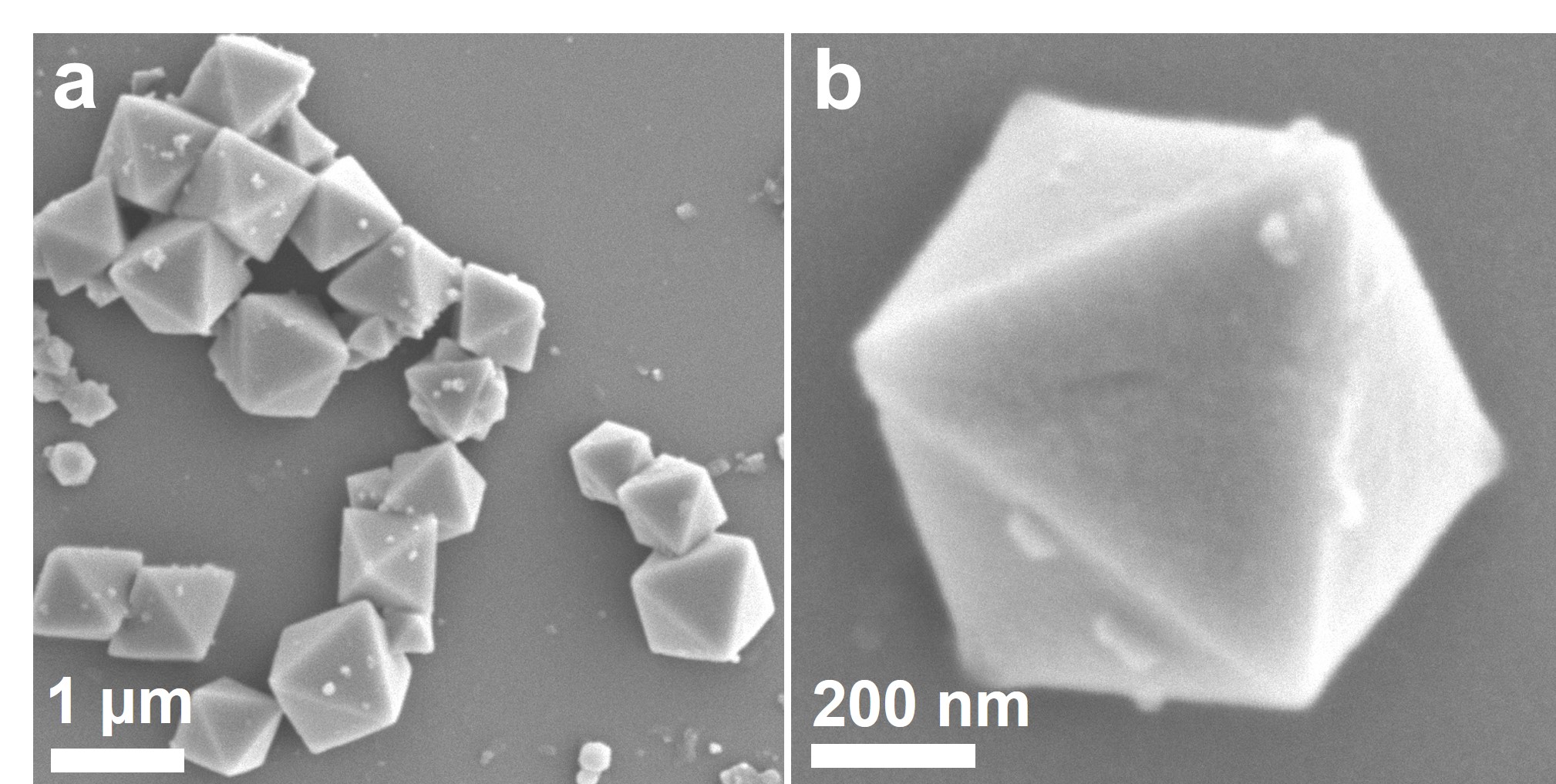


**Figure S2.** a, b) SEM images of UiO-66.





**Figure S3.** TGA curve of Ru-containing UiO-66 at N_2_ atmosphere.


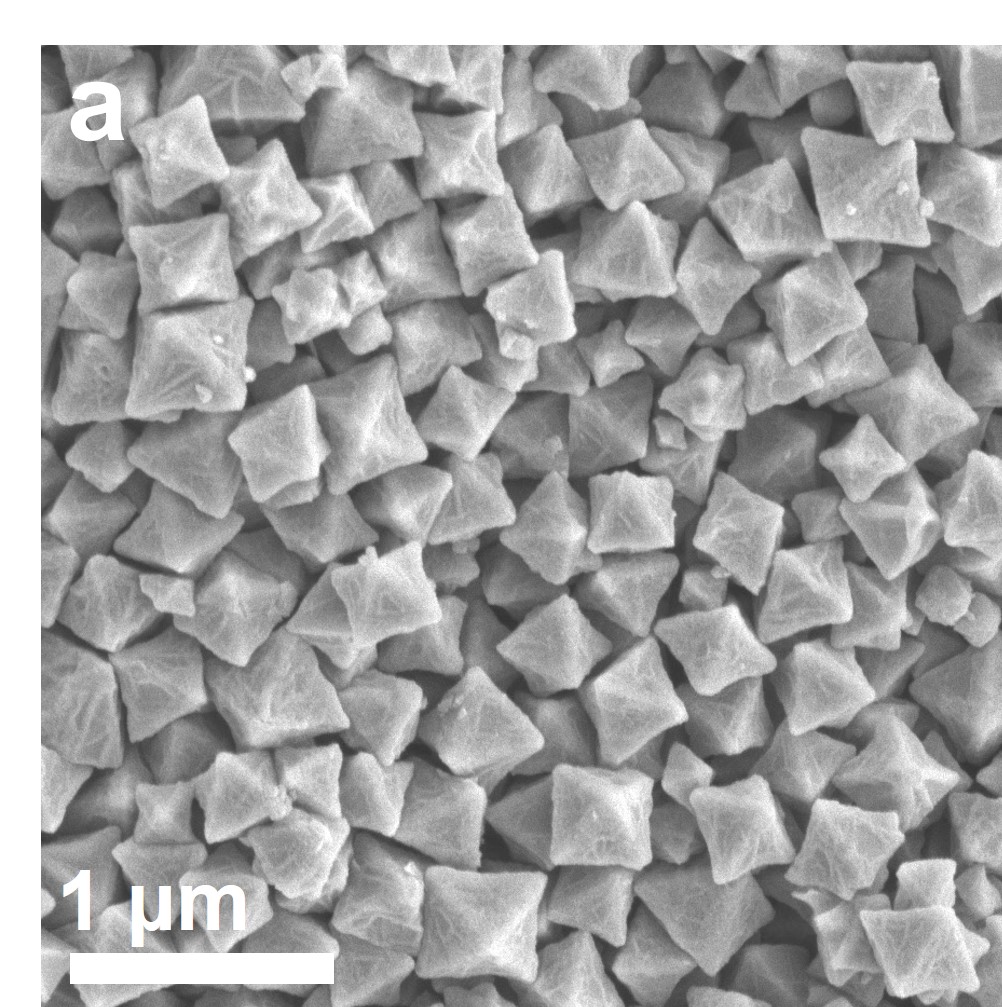


**Figure S4.** SEM image of Ru-SA-ZrO_2-x_/C.





**Figure S5.** Particle size distribution of Ru-SA-ZrO_2-x_/C.





**Figure S6.** STEM-EDS spectrum of Ru-SA-ZrO_2-x_/C catalyst.





**Figure S7.** XRD patterns of Ru-SA-ZrO_2-x_/Cs with different carbonization temperatures.





**Figure S8.** N_2_ adsorption-desorption isotherms of ZrO_2-x_/C and Ru-SA-ZrO_2-x_/C.





**Figure S9.** EPR spectra of ZrO_2-x_/C and Ru-SA-ZrO_2-x_/C.





**Figure S10.** XPS spectra of ZrO_2-x_/C and Ru-SA-ZrO_2-x_/C.


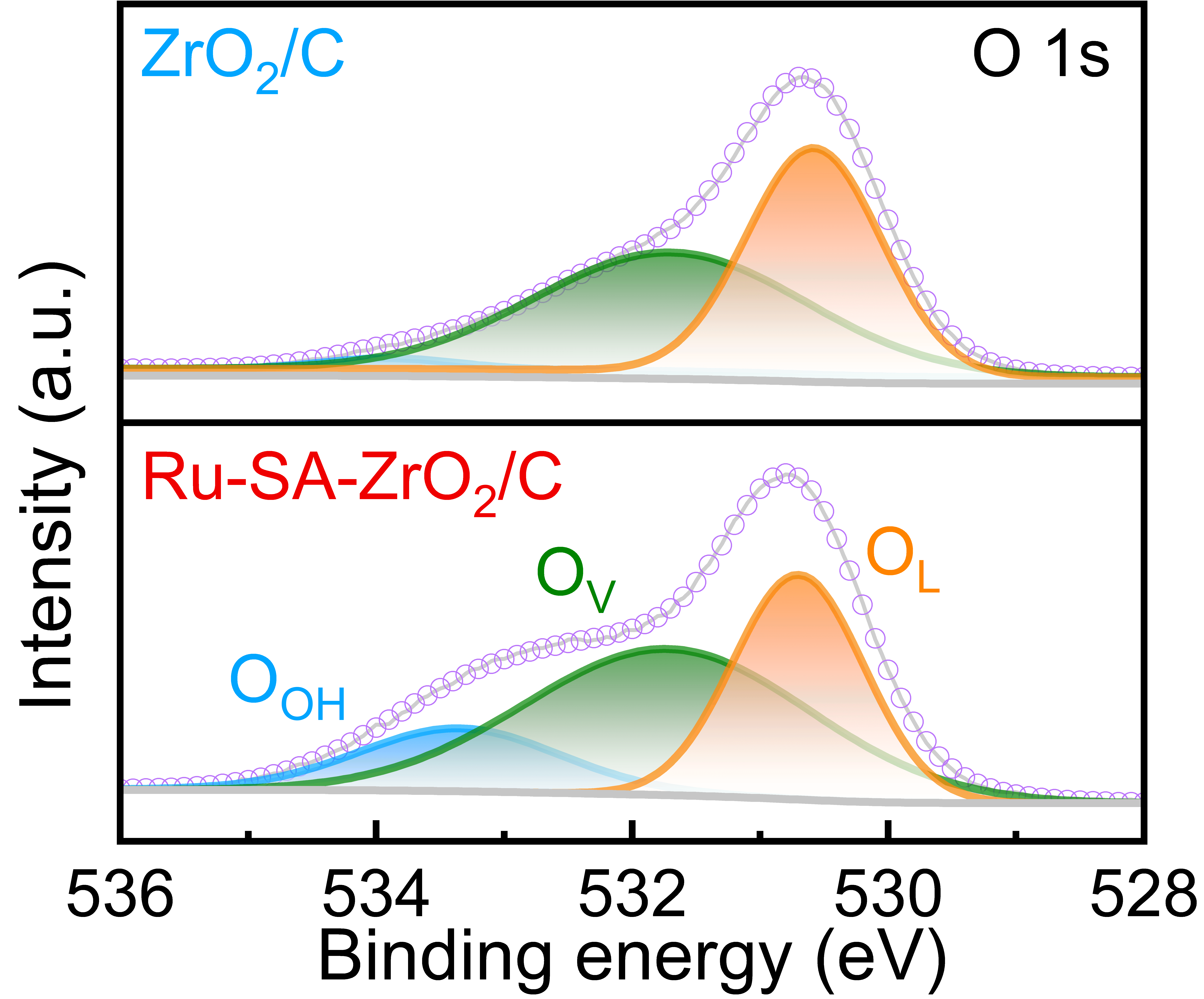


**Figure S11.** XPS spectra of the O 1s regions for Ru-SA-ZrO_2-x_/C and ZrO_2-x_/C, respectively.


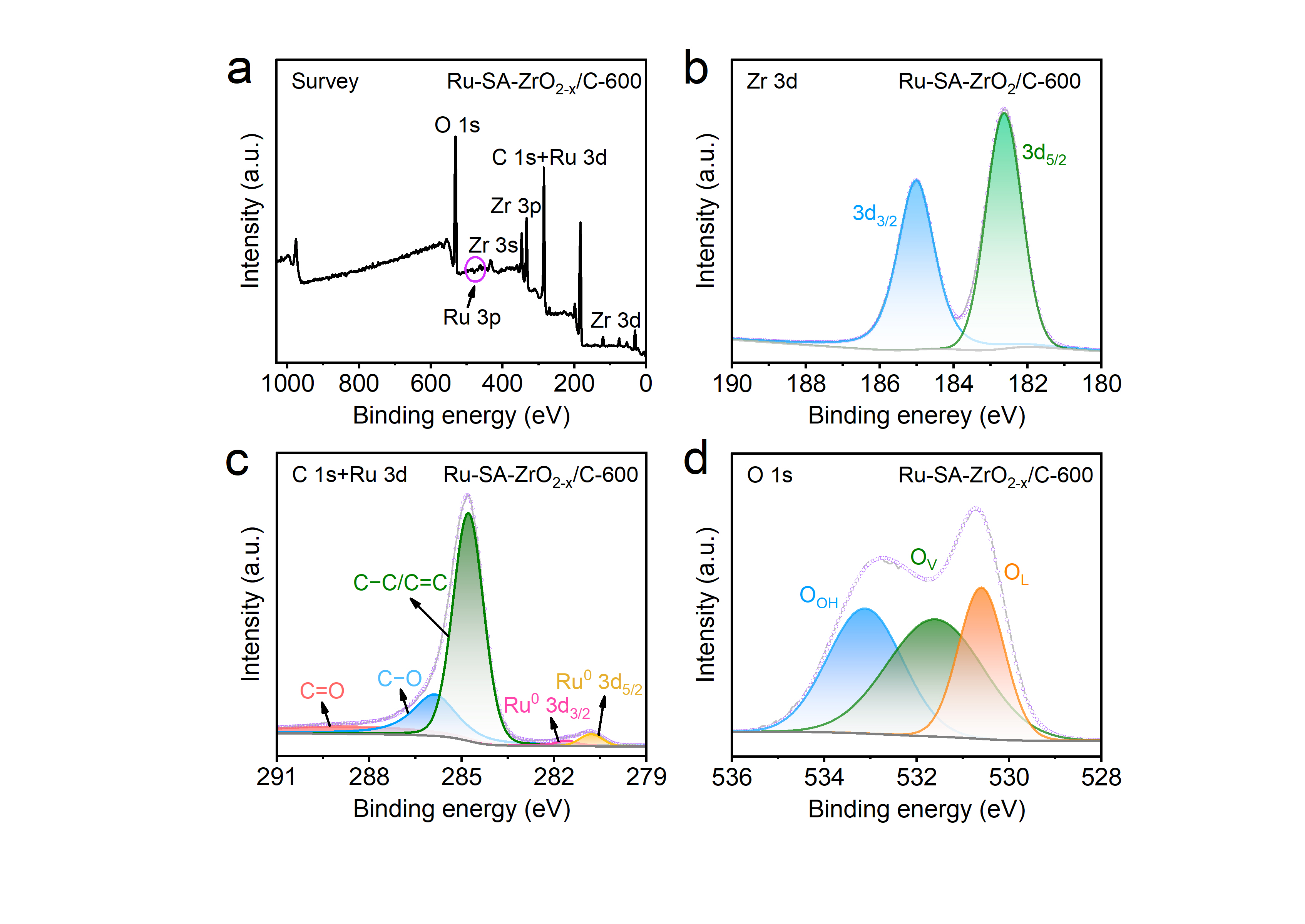


**Figure S12.** XPS spectrum of Ru-SA-ZrO_2-x_/C-600. XPS spectra of the b) Zr 3d, c) C 1s and Ru 3d, d) O 1s regions for Ru-SA-ZrO_2-x_/C-600, respectively.


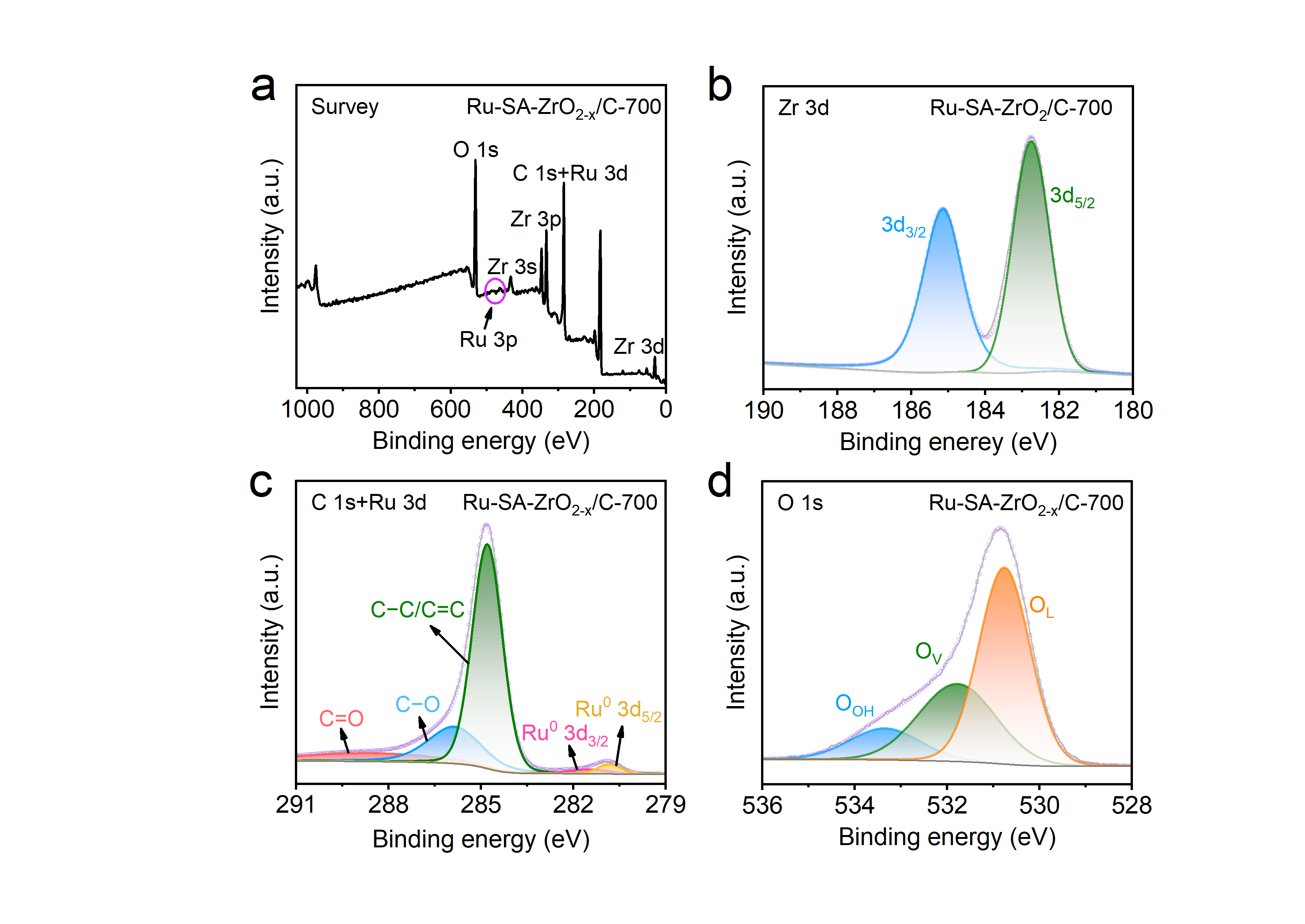


**Figure S13.** XPS spectrum of Ru-SA-ZrO_2-x_/C-700. XPS spectra of the b) Zr 3d, c) C 1s and Ru 3d, d) O 1s regions for Ru-SA-ZrO_2-x_/C-700, respectively.


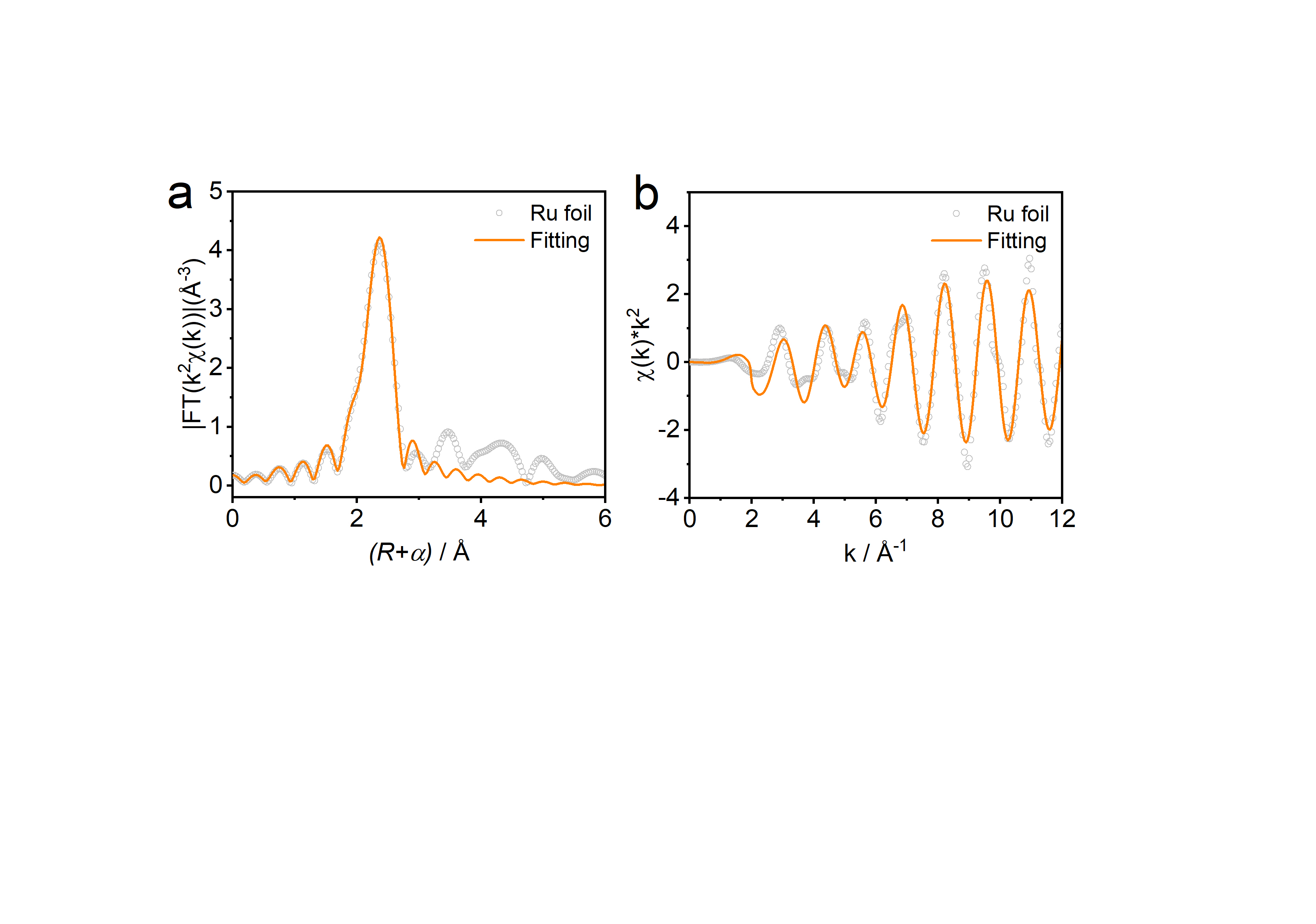


**Figure S14.** EXAFS fitting curves of Ru foil at the a) R space and b) K space.


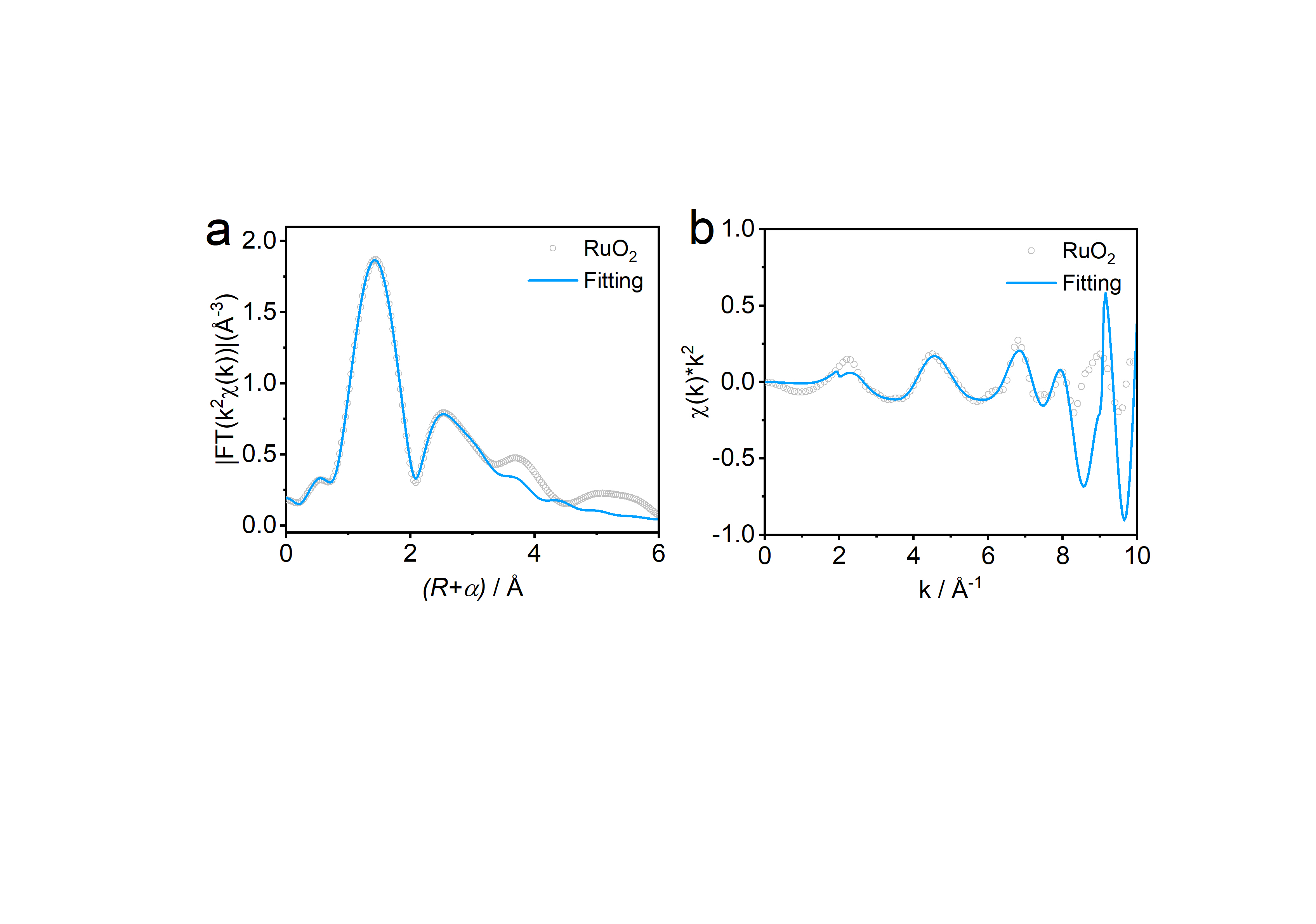


**Figure S15.** EXAFS fitting curves of RuO_2_ at the a) R space and b) K space.





**Figure S16.** The Koutecky-Levich plot of Ru-SA-ZrO_2-x_/C at an overpotential of 150 mV.





**Figure S17.** Polarization curves of Ru-SA-ZrO_2-x_/C in H_2_-saturated and Ar-saturated 0.1 M KOH with a scan rate of 5 mV s^−1^ at rotation rate of 1600 rpm.


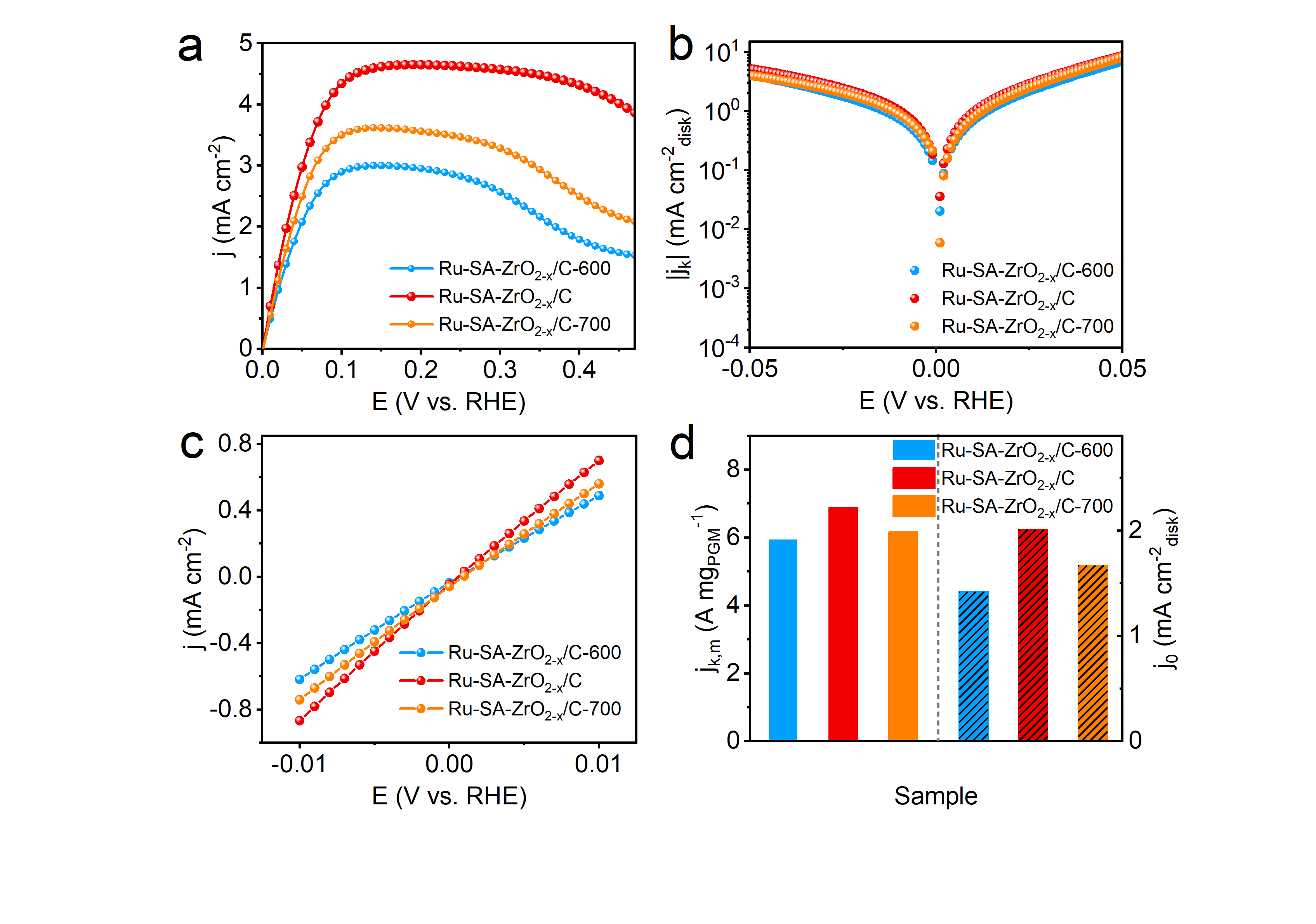


**Figure S18.** a) HOR polarization curves of Ru-SA-ZrO_2-x_/C-600, Ru-SA-ZrO_2-x_/C and Ru-SA-ZrO_2-x_/C-700 in H_2_-saturated 0.1 M KOH with scan rate of 5 mV s^−1^ at 1600 rpm. b) Tafel plots with Butler-Volmer fitting solid lines. c) Linear fitting curves in micro-polarization region (−10 to 10 mV). d) Mass activities at 50 mV and exchange current densities (j_0_) comparison of Ru-SA-ZrO_2-x_/C-600, Ru-SA-ZrO_2-x_/C and Ru-SA-ZrO_2-x_/C-700.


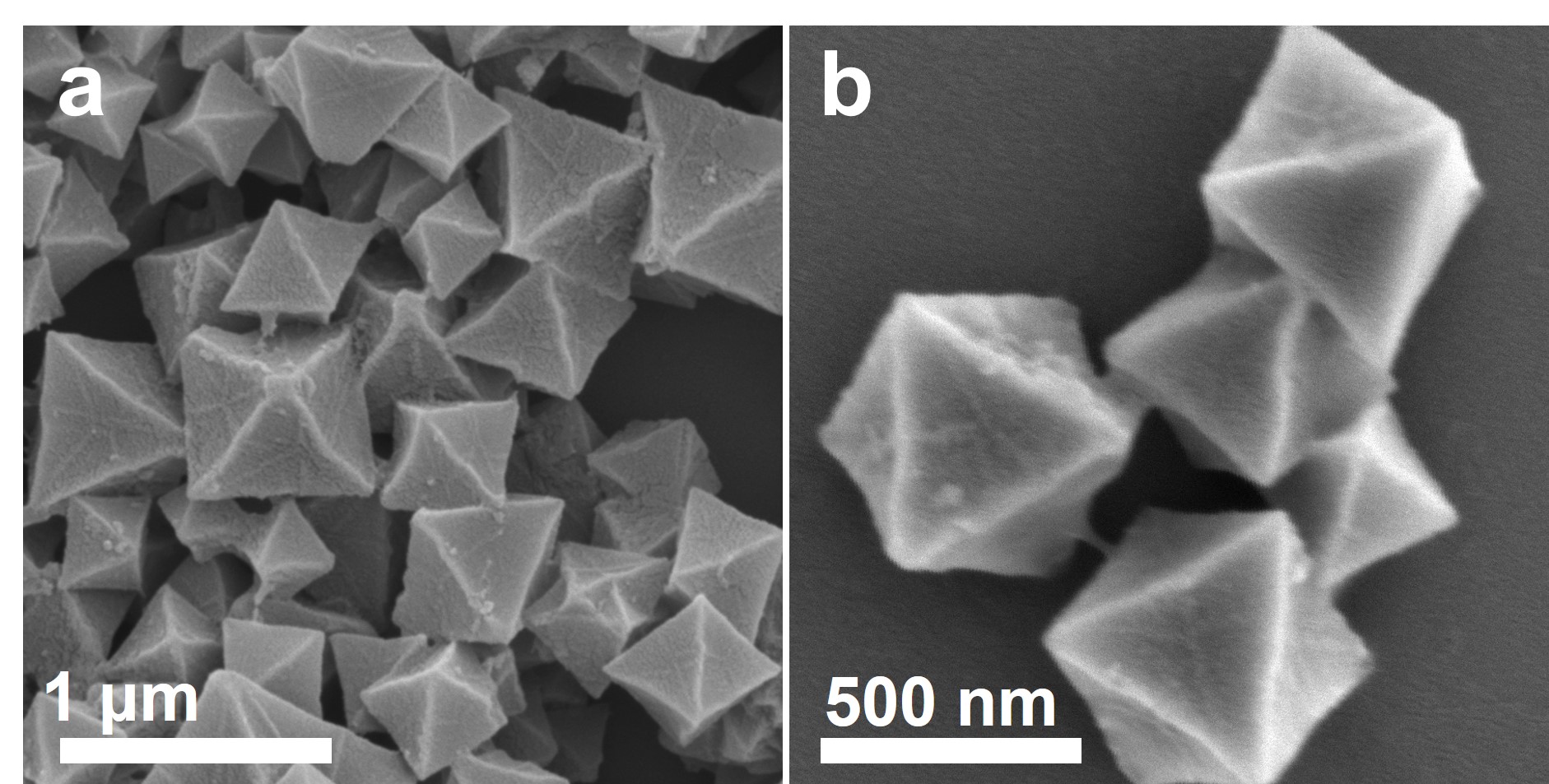


**Figure S19.** SEM image of Ru-SA-ZrO_2-x_/C after long-term stability test of 9500 seconds.





**Figure S20.** XRD patterns of Ru-SA-ZrO_2-x_/C and Ru-SA-ZrO_2-x_/C after long-term stability test of 9500 seconds.


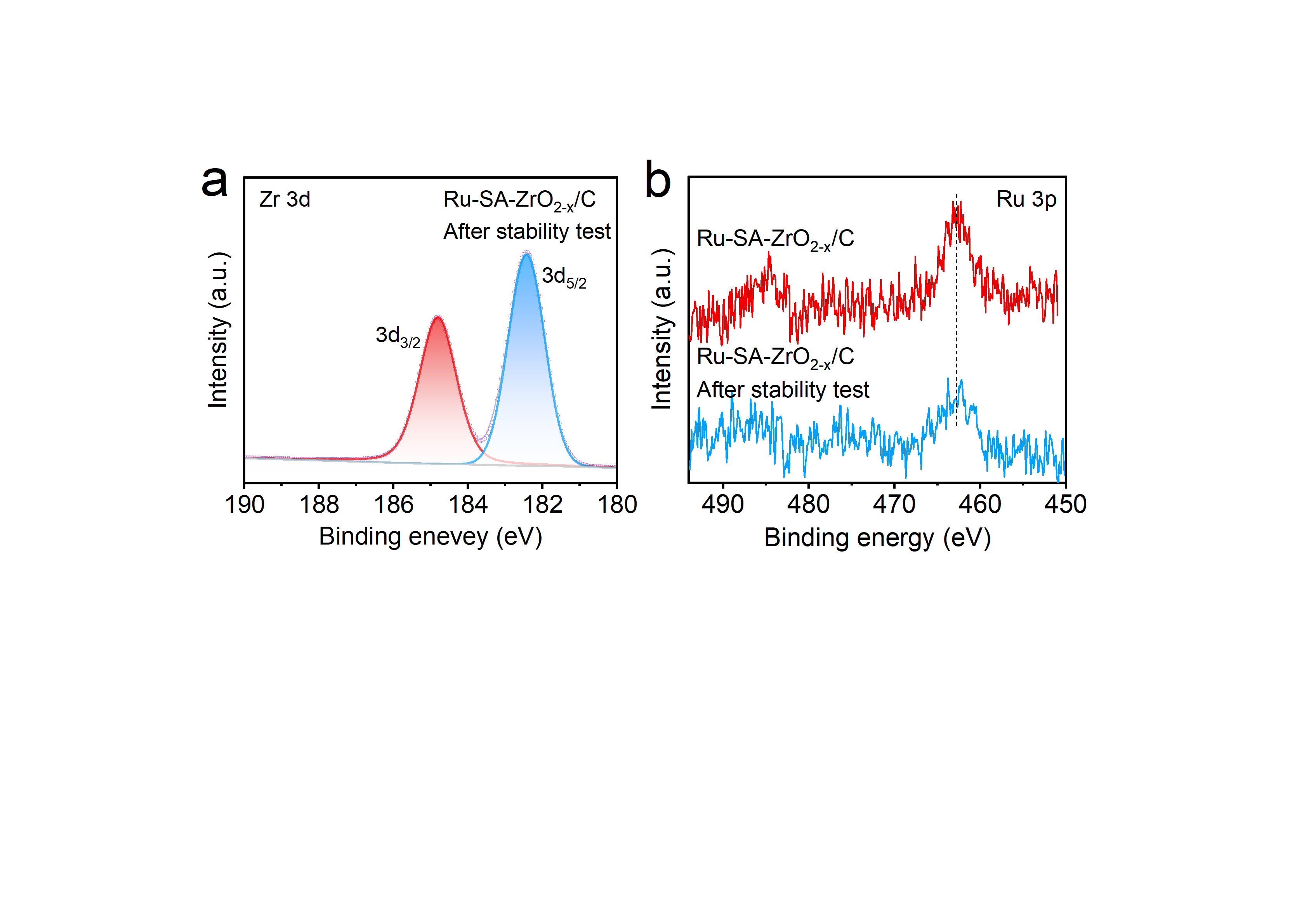


**Figure S21.** XPS spectra of a) Zr 3d and b) Ru 3p of Ru-SA-ZrO_2-x_/C after long-term stability test of 9500 seconds.


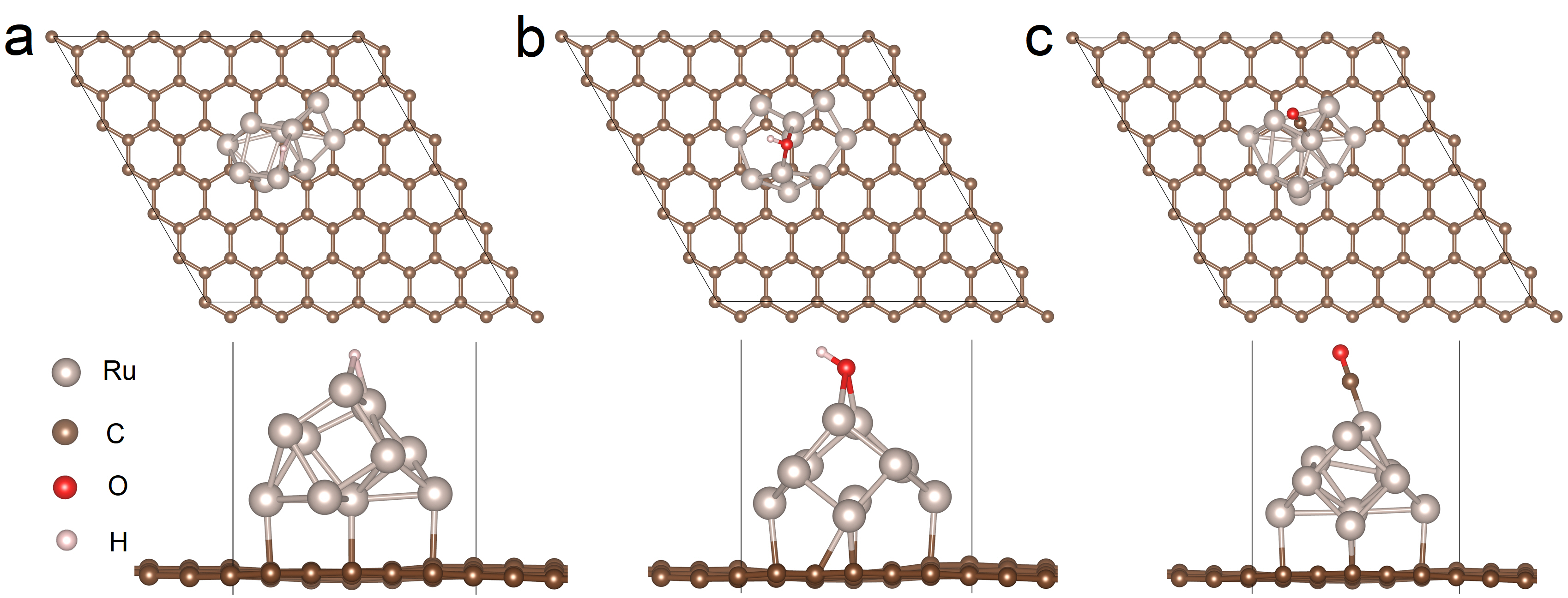


**Figure S22.** Optimized models for side and top views of a) H*, b) OH*, C) CO* adsorption on Ru cluster.


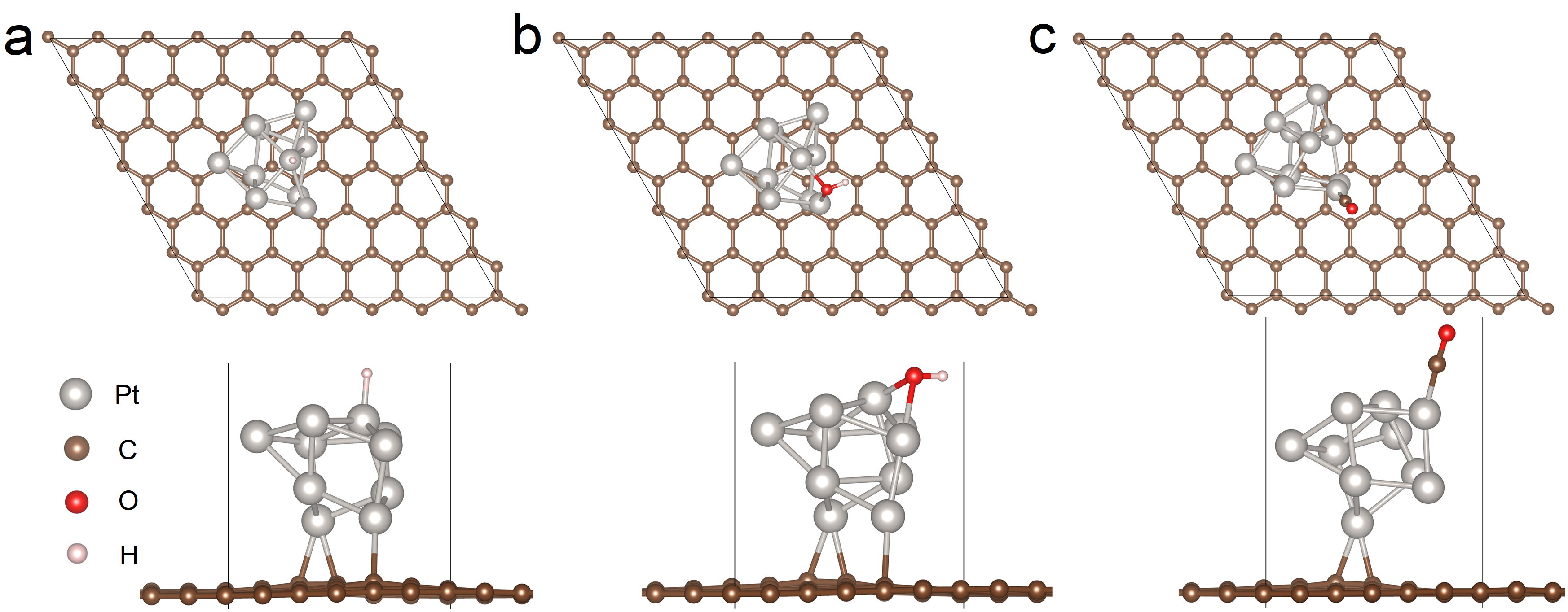


**Figure S23.** Optimized models for side and top views of a) H*, b) OH*, C) CO* adsorption on Pt/C.


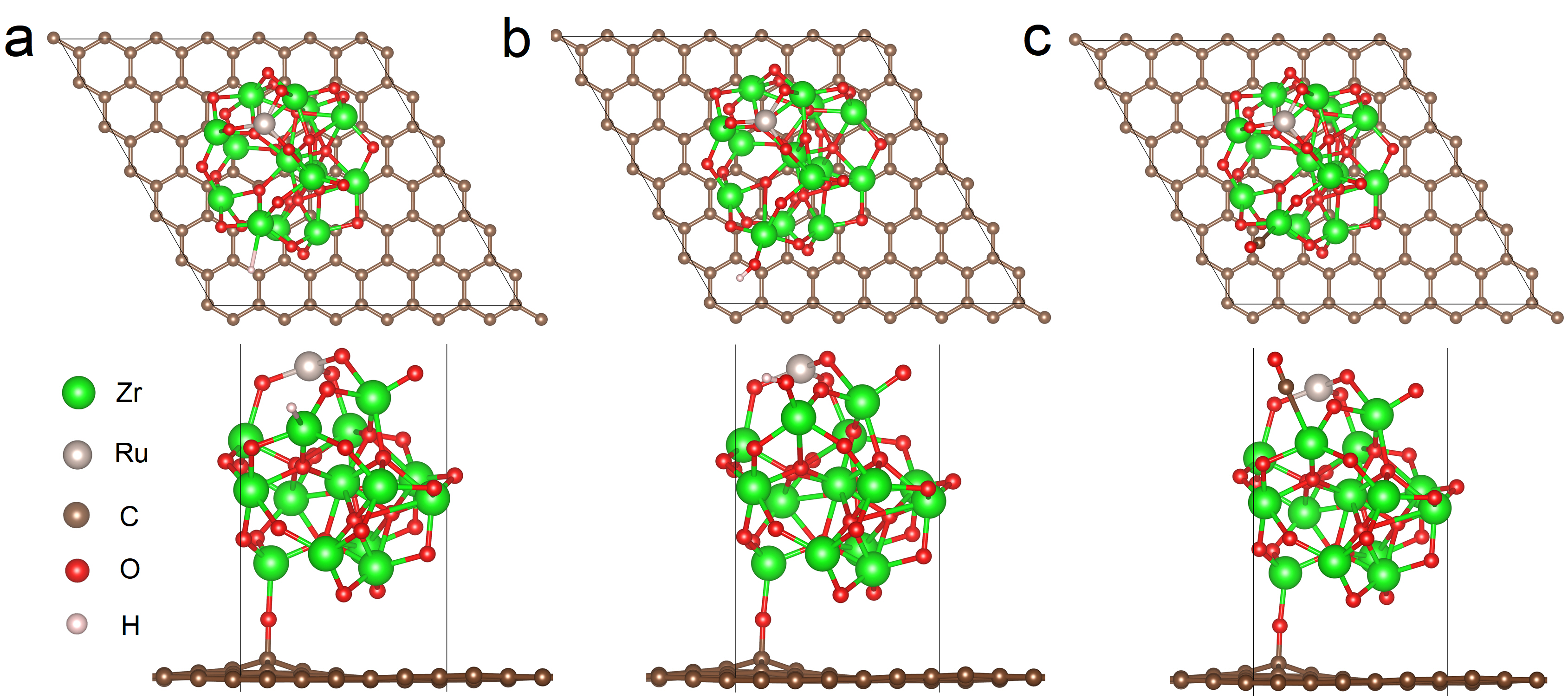


**Figure S24.** Atomic configuration of Ru-SA-ZrO_2-x_/C and a) H*, b) OH*, c) CO* adsorbed on Zr site on Ru-SA-ZrO_2-x_/C from both the side view and the top view.

**Table S1.** Inductive Coupled Plasma (ICP) results of prepared samples.

| **Samples** | **Ru-SA-ZrO_2-x_/C-600** | **Ru-SA-ZrO_2-x_/C** | **Ru-SA-ZrO_2-x_/C-700** |
| --- | --- | --- | --- |
| **Ru (%)** | 0.40 | 0.42 | 0.45 |

**Table S2.** Structural parameters extracted from the EXAFS fitting. R is the bond distance; CN is coordination number; σ^2^ is the Debye-Waller factor; ΔE_0_ is adjustable “muffin-tin zero”; R factor is goodness of fit. (S_0_^2^=0.96).

| **Samples** | **Path** | **N** | **R(****Å)** | **σ^2^/ Å^2^** | **ΔE_0_(eV)** | **R factor** |
| --- | --- | --- | --- | --- | --- | --- |
| **Ru foil** | Ru−Ru | 12 | 2.68 | 0.0035 | -5.7 | 0.014 |
|  | Ru−O | 6 | 1.97 | 0.0003 |  |  |
| **RuO_2_** | Ru−Ru1 | 2 | 3.14 | 0.0143 | -1.18 | 0.002 |
|  | Ru−Ru2 | 8 | 3.39 | 0.0116 |  |  |
| **Ru-SA-ZrO_2-x_/C** | Ru−O | 3.1 | 2.05 | 0.0003 | 4.47 | 0.013 |

**Table S3.** Comparison of HOR performance for Ru-SA-ZrO_2-x_/C with recently reported electrocatalysts.

| Catalysts | Metal loading (μg_metal_ cm^−2^) | Mass activity  (A mg^−1^_metal_) | Ref. |
| --- | --- | --- | --- |
| Ru-SA-ZrO_2-x_/C | 1.25 | 6.79 | This work |
| Mo-Pt/NC | 10 | 4.55 | 3 |
| V-S-Ru/C | 4.20 | 0.51 | 4 |
| Ir_1_Ru_3_ NWs/C | 29.0 | 3.35 | 5 |
| IO-Ru–TiO_2_/C | 25.48 | 0.91 | 6 |
| Ru/VOC | 18.47 | 3.44 | 7 |
| Sn-Ru/C | 6.26 | 1.79 | 8 |
| Ru/NiSe_2_ | 8.84 | 0.32 | 9 |
| RuNi/C | 10 | 2.33 | 10 |
| Ru-WC_x_ | 1.17 | 7.84 | 11 |
| IrMo_0.59_ NPs | 1.75 | 3.85 | 12 |

**References**

1. G. Kresse, J. Furthmüller, *Comput. Mater. Sci.* **1996**, *6*, 15−50.
2. J. P. Perdew, K. Burke, M. Ernzerhof, *Phys. Rev. Lett.* **1996**, *77*, 3865−3868.
3. M. Ma, G. Li, W. Yan, Z. Wu, Z. Zheng, X. Zhang, Q. Wang, G. Du, D. Liu, Z. Xie, Q. Kuang, L. Zheng, *Adv. Energy Mater.* **2022**, *12*, 2103336.
4. Q. Wu, W. Yang, X. Wang, W. Zhu, S. Lv, Y. Zhou, T. Chen, S. Liu, W. Li, Z. Chen, *Appl. Catal. B Environ.* **2023**, *335*, 122896.
5. B. Qin, H. Yu, X. Gao, D. Yao, X. Sun, W. Song, B. Yi, Z. Shao, *J. Mater. Chem. A* **2018**, *6*, 20374−20382.
6. J. Jiang, S. Tao, Q. He, J. Wang, Y. Zhou, Z. Xie, W. Ding, Z. Wei, *J. Mater. Chem. A* **2020**, *8*, 10168−10174.
7. P. Wang, Y. Yang, W. Zheng, Z. Cheng, C. Wang, S. Chen, D. Wang, J. Yang, H. Shi, P. Meng, P. Wang, H. Tong, J. Chen, Q. Chen, *J. Am. Chem. Soc.* **2023**, *145*, 27867−27876.
8. L. Wu, L. Su, Q. Liang, W. Zhang, Y. Men, W. Luo, *ACS Catal.* **2023**, *13*, 4127−4133.
9. Y. Feng, S. Lu, L. Fu, F. Yang, L. Feng, *Chem. Sci.* **2024**, *15*, 2123−2132.
10. T. Jiang, Z. Liu, Y. Yuan, X. Zheng, S. Park, S. Wei, L. Li, Y. Ma, S. Liu, J. Chen, Z. Zhu, Y. Meng, K. Li, J. Sun, Q. Peng, W. Chen, *Advanced Materials* **2023**, *35*, 2300502.
11. L. Wang, Z. Xu, C. H. Kuo, J. Peng, F. Hu, L. Li, H. Y. Chen, J. Wang, S. Peng, *Angewandte Chemie International Edition* **2023**, *62*, e202311937.
12. L. Fu, Y. Li, N. Yao, F. Yang, G. Cheng, W. Luo, *ACS Catal.* **2020**, *10*, 7322−7327.
